# Context-Specific Arousal During Resting in Wolves and Dogs: Effects of Domestication?

**DOI:** 10.3389/fpsyg.2020.568199

**Published:** 2020-11-24

**Authors:** Hillary Jean-Joseph, Kim Kortekaas, Friederike Range, Kurt Kotrschal

**Affiliations:** ^1^Department of Behavioral and Cognitive Biology, University of Vienna, Vienna, Austria; ^2^Wolf Science Center, Domestication Lab, Konrad Lorenz Institute of Ethology, University of Veterinary Medicine, Vienna, Austria

**Keywords:** heart rate, heart rate variability, dogs, wolves, domestication, dog-human relationship

## Abstract

Due to domestication, dogs differ from wolves in the way they respond to their environment, including to humans. Selection for tameness and the associated changes to the autonomic nervous system (ANS) regulation have been proposed as the primary mechanisms of domestication. To test this idea, we compared two low-arousal states in equally raised and kept wolves and dogs: resting, a state close to being asleep, and inactive wakefulness, which together take up an important part in the time budgets of wolves and dogs. We measured arousal via cardiac output in three conditions: alone, with a familiar human partner, or with pack members (i.e., conspecifics). Specifically, we compared heart rate (HR) and heart rate variability (HRV) of six wolves and seven dogs. As patterns of resting can vary adaptively, even between closely related species, we predicted that dogs would be generally more aroused than wolves, because living with humans may come with less predictable contexts than living with conspecifics; hence, dogs would need to be responsive at all times. Furthermore, we predicted that due to the effects of domestication, emotional social support by familiar people would reduce arousal more in dogs than in equally human-socialized wolves, leading to more relaxed dogs than wolves when away from the pack. Overall, we found a clear effect of the interactions between species (i.e., wolf versus dog), arousal state (i.e., resting or awake inactive) and test conditions, on both HR and HRV. Wolves and dogs were more aroused when alone (i.e., higher HR and lower HRV) than when in the presence of conspecifics or a familiar human partner. Dogs were more relaxed than wolves when at rest and close to a familiar human but this difference disappeared when awake. In conclusion, instead of the expected distinct overall differences between wolves and dogs in ANS regulation, we rather found subtle context-specific responses, suggesting that such details are important in understanding the domestication process.

## Introduction

Domesticated species differ systematically from their wild conspecifics (“domestication syndrome,” Darwin, 1859; Wilkins et al., 2014). As the primary domestication mechanism seems to be selection for tameness (Belyaev, 1979; Trut et al., 2004), domesticated animals have been suggested to be hypersocial – defined as “a heightened propensity to initiate social contact that often extends to members of others species” – toward other individuals, including humans, compared to their wild counterparts (vonHoldt et al., 2017). The dog-wolf model is a great system to investigate potential differences due to domestication. Dogs began to diverge genetically from wolves some 35,000 years ago (Frantz et al., 2016; Botigué et al., 2017). Domestication has affected the ecology and behavior of dogs (Axelsson et al., 2013; Miklósi and Topál, 2013; Range and Virányi, 2015; Marshall-Pescini et al., 2017; Kotrschal, 2018). Wolves are cooperative hunters and breeders which generally avoid close contact with humans (Mech and Boitani, 2003). Still, equally human-socialized wolves and dogs behave relatively similar in experimental situations. For example, they are attentive toward humans (Range and Virányi, 2013) and cooperate with them (Range et al., 2019b), greet familiar and unfamiliar humans (Hall et al., 2015; Ujfalussy et al., 2017), and like in dogs, their salivary cortisol decreases during training sessions (Vasconcellos et al., 2016). Human-socialized adult wolves maintain social bonds with their early caretakers and other familiar people (preprint; Wheat et al., 2020) and, hence, may benefit from their presence in stressful situations via emotional social support. Still, wolves may not depend on humans as much as dogs that are raised and kept in a similar way (Topál et al., 2005) because dogs usually live in human environments (Coppinger and Coppinger, 2001) and may be selected for attaching easily and strongly to their human caretakers (Palmer and Custance, 2008; Gácsi et al., 2013; Solomon et al., 2019).

Previous studies have shown a clear interaction between dogs’ physiology and their emotional bonds with humans. For example, kennel dogs exposed to a novel environment in the presence of their human caretaker showed no increase in glucocorticoids, which was in contrast to when they were tested alone or with a familiar dog in a novel environment (Tuber et al., 1996). A study of pet dogs found that when dogs were petted by their owner during a veterinary examination dogs’ heart rate and ocular surface temperature increased less than when facing examination alone (Csoltova et al., 2017). Furthermore, another study of pet dogs showed that dogs’ heart rate variability (HRV) generally increased in response to being pet by their owners (Katayama et al., 2016), which suggests that this is experienced as a positive and rewarding situation. In humans also, heart rate and HRV is linked to emotional responses (Porges, 1995; Appelhans and Luecken, 2006).

The autonomic nervous system (ANS) regulates the heart and other visceral functions (Shields, 1993), including the expression of emotions in the social context (Porges, 2003). As domesticated animals differ from their wild ancestors mainly in their emotional responses to human contexts, domestication may have affected ANS modulations, the more as the neural crest hypothesis suggests that ‘initial selection for tameness leads to a change in the neural-crest-derived tissues’ (Wilkins et al., 2014), which includes the ANS. In turn, this leads to the changes observed trough all domesticated species, the so called “domestication syndrome” (Darwin, 1859; Wilkins et al., 2014).

The modulation of the ANS can be estimated via cardiac activity (Porges, 1995, 2001, 2003, 2009; Rajendra Acharya et al., 2006; Kreibig, 2010). Useful parameters are HR, i.e., the number of heartbeats per time unit, and HRV, i.e., the variation over time of the period between consecutive heartbeats (R-R intervals). While both physical activity and mental states modulate HR (Visser et al., 2002; Maros et al., 2008), HRV is less dependent on physical activity than HR, but generally decreases via psychological stress and increases during relaxation (Luque-Casado et al., 2013). A way to avoid the effect of physical activity on ANS modulation and thus, cardiac outputs, and test the emotional effect of social support by a social partner on wolves and dogs is to compare their arousal at rest.

Rest is considered as an intermediate state of the sleep-wakefulness continuum (Campbell and Tobler, 1984) defined as, “a state of reduced activity without the loss of consciousness or greatly reduced responsiveness” (Lesku et al., 2006; Siegel, 2008). At rest, parasympathetic activity increases, resulting in a comparatively low HR and high HRV. In addition, respiration deepens in association with a sinusoid pattern of HR, which is caused by the rhythmic breathing of the animal. This is called respiratory sinus arrhythmia (RSA, Oken et al., 2006). As RSA is present in both wolves (Kreeger et al., 1990) and dogs (Hamlin et al., 1966), we used it as a marker of a deep state of relaxation, i.e., resting (Kortekaas and Kotrschal, 2019). In the present study, we focused on the resting state but we also included a state in which an animal is more aroused and alert than during rest, i.e., the awake inactive state in which an animal is paying close attention to its environment (Oken et al., 2006) but is still physically inactive. With the term “arousal,” we integrate correlated mental and physiological states; low arousal such as during rest is equivalent to decreased consciousness toward environmental stimuli, with relatively low HR and high HRV.

Not much is known about dog resting patterns; they probably change with age, environment, and social context as it has been observed with sleep patterns. For example, living conditions affected the length of dog sleep cycles and the time they spent sleeping (Adams and Johnson, 1993) as shelter dogs slept more but with shorter sleep cycles than dogs living with owners. Interestingly, if more than one dog lived in the same household, the dogs tended to show asynchronous sleep-wake cycles (Adams and Johnson, 1993), meaning that at least one of them was awake at any time. Other studies have shown that after an active day, dogs are drowsier earlier and sleep more (Bunford et al., 2018) and older dogs sleep more during the day and less at night than younger dogs (Takeuchi and Harada, 2002; Bódizs et al., 2020). Also, the frequency of sleep spindles (i.e., a burst of brain activity) has been shown to vary with age, physical activity, social pre-sleep activity, sex, and reproductive status (Kis et al., 2014, 2017; Iotchev et al., 2019), factors which also have been shown to affect the HR and HRV of dogs before falling asleep (Varga et al., 2018). In adaptation to different ecologies and lifestyles, sleep patterns may vary substantially, even among closely related species (Siegel, 2005; Lesku et al., 2008; Aulsebrook et al., 2016). For example, birds (gadwall and black-tailed Godwit) had their eyes open for longer periods of time during rest/sleep when in large groups or in the center of the flock (Gauthier-Clerc et al., 2000; Dominguez, 2003). Whereas in yellow baboons, individuals in smaller groups were resting higher above ground than individuals in bigger groups (Stacey, 1986). Socialized wolves, were found to be more relaxed than dogs and have lower HR and higher HRV during periods of rest and inactive wakefulness (Kortekaas and Kotrschal, 2019). This has been suggested to be a specific adaptation of dogs for living in a human environment, which is presumably less predictable than the pack environment of wolves and hence, would necessitate a greater basic alertness. However, in this study, an unfamiliar human was present during the recording of the animals’ behavior, which might have influenced the results.

In our present study we assessed the effect of domestication on the modulation of dogs’ ANS by comparing wolves’ and dogs’ ANS modulation in three different social conditions: alone, with a familiar human, and with the other pack members. Similar to Kortekaas and Kotrschal (2019), we investigated two behavioral states with minimal physical activity and sensory stimulation: resting (animal is lying immobile and eyes closed) and inactive wakefulness (animal is lying with its head in an upward position with the eyes open). We compared cardiac output in similarly raised and kept, and therefore fully comparable, group-living wolves and dogs. We selected periods of respiratory sinus arrhythmia for analysis, as this is indicative of rest (Kortekaas and Kotrschal, 2019).

As human-socialized wolves form social bonds with familiar humans, we expected that emotional support by humans would modulate their ANS. However, due to dogs’ adaptation to the human environment during domestication, the proposed emotional support effect should be more pronounced in dogs than wolves. Different predictions can be generated from the major dog domestication hypotheses. If selection for tameness as primary mechanism of domestication (Belyaev, 1979; Wilkins et al., 2014) shaped dogs’ ANS’s modulation, we predicted they would be generally more relaxed (i.e., less reactive to stressful events and with lower HR and higher HRV) than their wild ancestors (Darwin, 1868; Price, 1999; Hare et al., 2012). Based on the hypersociality hypothesis (vonHoldt et al., 2017), we expected that dogs would benefit more (i.e., be more relaxed at rest and with lower HR and higher HRV compared to a control condition where the animals are alone) in the presence of a human and possibly also in the presence of conspecifics than wolves. Alternatively, the intention to interact with the familiar person or pack member – due to the proposed higher sociability of dogs as compared to wolves – might result in increased arousal (higher HR and lower HRV). However, this was not expected to be the case once the animals has settled down to rest.

In contrast to the precedent domestication hypotheses, the canine cooperation hypothesis (Range and Virányi, 2015) suggests that dogs’ social and cooperative skills toward humans are not a by-product of domestication but rather a direct wolf heritage originating from the wolves’ social orientation toward pack members. This hypothesis suggests that during domestication dogs shifted their cooperative orientation to humans. Based on this hypothesis we predicted that wolves as well as dogs would relax in the presence of both a familiar human and a conspecific pack member. This is in alignment with the deferential hypothesis (Range et al., 2019b), which predicts that dogs should benefit more from the human presence than wolves, dogs should gain a greater support effect from humans (i.e., lower HR and higher HRV with a human compared to being with other dogs) whereas wolves would gain great support effect from conspecifics (i.e., lower HR and higher HRV with their conspecifics). In contrast to the hypersociability hypothesis (vonHoldt et al., 2017), the deferential hypothesis would not predict that dogs can benefit more from the presence of other dogs.

## Materials and Methods

### Ethical Approval

This research was approved by the institutional ethics committee at the University of Veterinary Medicine, Vienna, in accordance with GSP guidelines and national legislation (ETK-11/11/2018).

All study animals were housed at the Wolf Science Center (WSC) located in the Game Park Ernstbrunn in Austria. Their participation in the experimental sessions was voluntary. If they were not motivated to leave their home enclosure, the session was canceled and repeated on a different day. In nine cases, trials had to be repeated for this reason. If the subject did not rest in the test enclosure during the session, the condition was repeated on another day, in total this situation happened 53 times. If they did not rest in five sessions, the subject was tested in its home enclosure while its pack mates were brought to the test enclosure (*n* = 2). Via these adjustments we also achieved a homogenous motivational basis for our experiments. Only animals in a positive/relaxed mood would participate, because temporarily wary or anxious animals would not leave their enclosure to participate. All animals at the WSC are well habituated to being shifted to and from their home enclosure and also to experimental procedures.

### Subjects

Subjects were six wolves, *Canis lupus* (three males and three females) and seven dogs, *Canis lupus familiaris* (four males and three females; see Table 1). All wolves and dogs were born in captivity and were hand-raised from 10 days old by humans following a standardized procedure to produce trustful and workable partners for research (Klinghammer and Goodman, 1987). At 5 months of age they were integrated into preexisting conspecific packs. For more details on the raising of the animals see Range and Virányi (2014).

**TABLE 1:** List of the subjects.

Individual	Species	Sex	Date of birth	Weight*	Pack size
Amarok	Wolf	♂	4.04.2012	39.82	2
Aragorn	Wolf	♂	4.05.2008	48.50	3
Chitto	Wolf	♂	4.04.2012	46.72	2
Shima	Wolf	♀	4.05.2008	39.50	2
Tala	Wolf	♀	4.04.2012	39.15	2
Yukon	Wolf	♀	2.05.2009	37.82	3
Enzi	Dog	♂	2.04.2014	29.00	4
Gombo	Dog	♂	21.03.2014	28.67	2
Hiari	Dog	♂	21.03.2014	24.13	3
Imara	Dog	♀	21.04.2014	21.39	3
Meru	Dog	♂	1.10.2010	24.18	2
Panya	Dog	♀	2.04.2014	25.20	4
Zuri	Dog	♀	24.05.2011	20.80	4

*Weights displayed here are the weights of the subjects over the three testing days in kilograms.

All animals were kept in small groups in outside enclosures ranging between 2,000 and 8,000 m^2^ in size with natural landscape including trees, bushes, shelters, and natural objects such as stones, branches, and tree trunks. The subjects were between 5 and 11 years of age when tested – wolves: median (range) = 7 (6–10); dogs: median (range) = 4 (4–8) and had between 20.8 and 48.9 kg – wolves: median (range) = 40 (37.5–48.9); dogs: median (range) = 24.5 (20.8–35). The wolves were fed with carcasses of deer, rabbit, or chicken 3–4 times a week, while the dogs were fed with commercial dog food daily. As the dogs could not be provided carcasses, like the wolves, the dogs were regularly provided food enrichment, such as small pieces of deer, rabbit, or chicken, to make wolf and dog feeding as similar as possible. Water was available *ad libitum* to all wolves and dogs, including during training and test situations. Wolves and dogs had the same amount of contact with humans and both received veterinary and obedience training from puppyhood and cooperated in a number of behavioral tests on a weekly basis. As a result, all animals were accustomed to participating in research while separated from their pack members.

### Data Collection

Overall, we tried to keep our methods as similar as possible to Kortekaas and Kotrschal (2019). Heart rate (HR) was measured via the Polar^®^ RS800CX system designed for human usage. The accuracy of the Polar system has been validated for dogs via a comparison with a conventional electrocardiogram (ECG; Jonckheer-Sheehy et al., 2012; Essner et al., 2013, 2015). The system consists of a chest belt with electrodes, which are fastened around the animals’ chest behind the shoulders. From there the data are sent to a watch-like data logger attached to a neck collar. As the belt was designed for humans, the fur of the wolves and dogs impedes the belt electrodes. Hence, the fur under the electrodes in the belt was wetted with 70% ethanol to enhance signal conductivity. The entire procedure was trained beforehand via positive reinforcement.

HR data were first checked for the presence of respiratory sinus arrhythmia as an indicator of deep rest (i.e., a sleep-like condition). Video recordings of these sessions were coded with Solomon Coder©. HR and behavioral data were manually synchronized. Specifically, when the watch started recording data, the experimenter said loudly “start,” which was used as a signal for synchronizing the video and the HR recording. For the resting condition, all HR data showing a respiratory sinus arrhythmia pattern and the matching resting behaviors was kept. For analyses, HR and HRV recording are required to be the same length of time to be comparable (von Borell et al., 2007). Most of our animals rested for more than 80 s but for one wolf (Amarok resting with a familiar human) and one dog (Enzi resting alone) 80 s was the shortest maximum time they spend resting. Hence, we selected 80 s HR strands for analysis and all bouts shorter than 80 s were excluded from further analyses. One resting bout was randomly selected per animal and conditions. For the inactive wakefulness, no specific HR pattern has been described (in analogy to RSA during rest), hence we selected any strand of HR data longer than 80 s that corresponded to awake but inactive behaviors (i.e., laying down immobile with eyes open). If the animal moved (i.e., changed the position of its body or its head) or closed its eyes for more than a blink (i.e., eyes closed for more than 1 s and opening again, 1 s corresponded to five frames on Solomon Coder) the HR strand was discarded. One strand of HR per animal and activity (i.e., resting or inactive wakefulness) was randomly selected. As we only had one HR recording per animal and condition, we avoided selecting multiple strands of the same activity (i.e., resting or inactive wakefulness) to avoid dependent data points.

HR measurements collected with the Polar system can contain artifacts, leading to the need for editing (von Borell et al., 2007). Accordingly, the HRs measured were corrected using the algorithm-supported visual error correction (AVEC) method (Schöberl et al., 2015), applying a confidence interval for the outliers of 95%. HR measurements with more than 5% of errors were excluded. Heart rate variability (HRV) in this study was expressed as the root mean square of successive differences (RMSSD), normally used for short-term HRV analysis (for RMSSD details see von Borell et al., 2007). Mean HR and RMSSD were calculated with Kubios©. Resting HRs and awake inactive HRs were taken from the same recording but were not time adjacent.

### Procedure

The experimental sessions were conducted during a quiet period of the day when the animals were resting (normally between 12 am and 2 pm). Depending on the condition, the focal subject was taken out of the pack and brought to a test enclosure with some distance to its home pack or was accommodated in the shifting system immediately adjacent to the pack enclosure (i.e., the subject was only separated from the pack mates by a single wire mesh). Before the onset of the experimental phase, an animal trainer applied the Polar-belt to the subject for the recording of the HR. During the test period (1 h), no human was present around the enclosure (i.e., keeping away from the enclosure and out of sight of the animal tested) except for the human company condition. Each session was recorded with one or two cameras (depending on the size and configuration of the enclosure).

The animals were tested in the following three conditions: (1) alone: the subject was alone in its enclosure; (2) human company: The subject was alone in its enclosure while a familiar human was sitting just outside the fence of the enclosure (minimal distance 50 cm). The subject was free to approach the human or to stay away. The familiar human was instructed to not interact with the subject, but instead was reading a book or working on a laptop; (3) conspecific company: The subject and its pack members (1–3) stayed in visual contact during the test hour but were separated by a fence.

We analyzed cardiac outputs in two different behavioral conditions, resting and inactive wakefulness. We used the same behavioral criteria as Kortekaas and Kotrschal (2019) to define two conditions.

1. Resting: The body touching the ground either with caudal, dorsal, or lateral side. The position of the paws varies, e.g., folded (under body) or stretched out. The head is in a downward position, either lying on paws, ground, or tucked under the body. The eyes are generally closed but may repeatedly open and close (peeking). Parts of the body occasionally twitching.
2. Inactive wakefulness: The subject is awake, body touching the ground either with caudal, dorsal, or lateral side. The position of the paws varies, e.g., folded (under body) or stretched out. The head is in an upward position and can be moved around. The eyes are open, but increased blinking can occur.

### Statistical Analyses

All models were fitted in R (version 3.6.1; R Core Team, 2019) using the function lmer of the R package lme4 (version 1.1-21; Bates et al., 2014). To test whether the cardiac parameters would differ depending on species, activity of the subject, and condition of the test, the response variables “mean” HR and RMSSD (a common measure for HRV) were both analyzed in separate linear mixed effect models (LME, Baayen, 2008). Species (wolf or dog), activity (resting or inactive wakefulness), condition of the test (alone, with a human, or with conspecifics) included as fixed effects factors. We also included in the model a three-way interaction between species, activity and condition (and also all three two-way interactions this encompasses) in order to understand how cardiac outputs changed as a function of activities and conditions and how these differences in cardiac outputs varied between wolves and dogs. To control for the effects of temperature, body mass, age, and sex, these factors were also included as fixed effects. Subject identity was included as a random intercept to account for individual differences and to avoid pseudo replication, as all subject were tested in each condition. To keep type I error rates at the nominal level of 5%, we included random slopes of condition and activity and also the correlation parameters among the random intercept and random slopes terms of the HR model (Schielzeth and Forstmeier, 2009; Barr et al., 2013). However, we chose to exclude those correlations from the HRV model because many of them were estimated to be close to 1 or −1 which is indicative of them to be unidentifiable (Matuschek et al., 2017). This led to an only moderate decrease in model fit (HRV model with correlations: logLik = −440.3498 (*df* = 32) and HRV model without correlations: logLik = −446.6024 (*df* = 22). Body mass, age, and temperature were z-transformed (to a mean of zero and a standard deviation of one). Activity, condition, and species were manually dummy-coded (i.e., the categorical predictors were replaced by one or several dummy variables, one for each level of the factor except its reference category, each consisting solely of 0 and 1 s to facilitate model computation) and then centered to a mean of zero before including them in the random slopes in the model.

We checked whether the residuals were normally distributed and homogeneous by visually inspecting a qqplot and the residuals plotted against fitted values. Both indicated no obvious deviations from these assumptions. We checked for model stability by excluding subjects one at a time from the data and comparing the model estimates derived for these subsets of the data with those derived for the full data set. Both models were unstable for the factor species (see Supplementary Tables 1, 2). To check for potential collinearity issues, we inspected Variance Inflation Factors (VIF, Field, 2005) which we derived using the function VIF of the R-package car (Fox and Weisberg, 2018), applied to a standard linear model excluding the random effects and interactions. This revealed that species and body mass were slightly collinear with a VIF of 11.57 and 10.21, respectively. However, there was considerable variation of body mass within both species and, hence, the results obtained for these two predictors should not be distorted by collinearity among them.

To avoid cryptic multiple testing and keep type I error rate at the nominal level of 0.05 (Forstmeier and Schielzeth, 2011) we tested the significance of the full model as compared to the null model (comprising only age, body mass, sex, temperature, and the random effects) by means of a likelihood ratio test (R function anova with argument test set to “Chisq”; Dobson and Barnett, 2018). To allow for a likelihood ratio test we fitted the models using maximum likelihood (rather than Restricted Maximum Likelihood; Bolker et al., 2009). *P*-values for the individual effects were based on likelihood ratio tests comparing the full with the respective reduced models (Barr et al., 2013; R function drop1).

The sample size for both these models was 73 observations made on 13 individuals (seven dogs, six wolves). Six data points were missing as the animals did not display the behaviors measured in this study (rest/inactive wakefulness). Four data points were included despites displaying bad RSA pattern to enhance model stability (Meru alone, Zuri alone and with human company, Hiari alone).

## Results

### Mean Heart Rate

Overall, species, activity, and condition had a clear effect on HR (full-null comparison likelihood ratio test: χ^2^ = 57.22, *df* = 11, *P* < 0.001). More specifically, we found that the interaction between species, activity, and condition had an effect on HR (χ^2^ = 10.60, *df* = 2, *P* = 0.005; Table 2) and that the HR differences between dogs and wolves varied depending on the combination of test conditions and activities.

**TABLE 2:** Results of the HR Model.

	Estimate	SE	χ^2^	F	P^1^
Intercept	97.245	6.440			
Species (0, dog; 1, wolf)	–25.237	9.604			
Human	9.908	4.123			
Conspecifics	3.002	5.315			
Activity (0, awake; 1, rest)	–11.773	3.560			
Body mass^2^	10.706	3.585	5.187	1	**0.023**
Temperature^2^	–2.027	1.016	3.378	1	0.066
Age^2^	15.248	2.402	14.648	1	**<0.001**
Sex (0, F; 1, M)	–5.251	2.470	3.015	1	0.082
Wolf:Human	–21.823	6.053			
Wolf:Conspecifics	–16.729	7.465			
Wolf:Rest	–7.956	4.942			
Human:Rest	–9.627	4.448			
Conspecifics:Rest	–7.030	4.361			
Wolf:Human:Rest	22.889	6.490	10.601	2	**0.005**^3^
Wolf:Conspecifics:Rest	10.030	6.185			

*Statistically significant p-value are in bold. ^1^Not indicated in the case where p-value had a limited interpretation. ^2^Predictors were z-transformed to a mean of zero and a standard deviation of one; original means (SD) were weight: 32.98 (9.49) kg, temperature: 22.41 (7.18)°C and age 2440.23 (801.93) days. ^3^Overall test of the three-way interaction between species, activity and conditions.*

**FIGURE 1:**
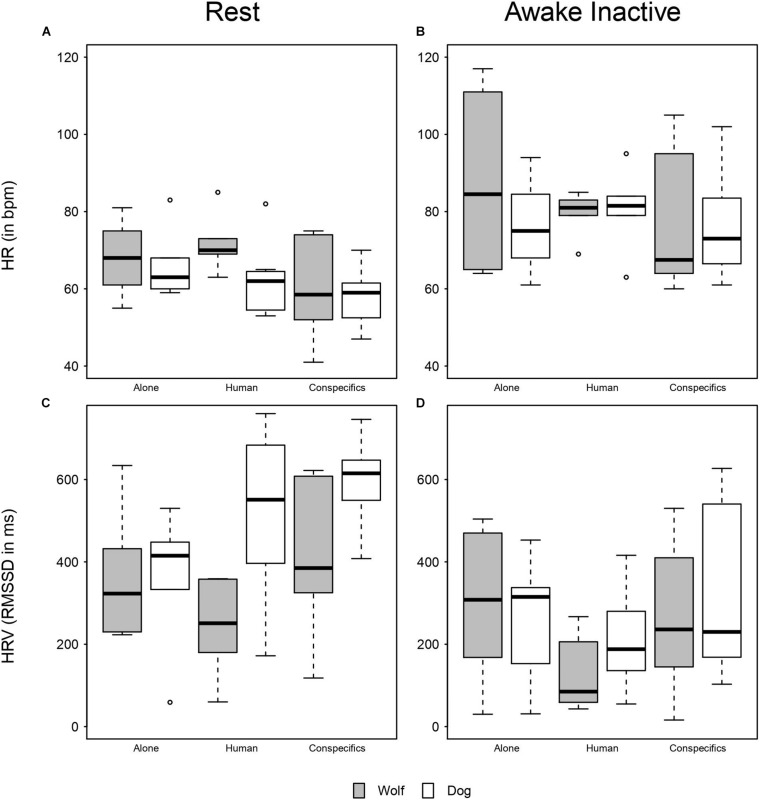
Boxplot of mean HR and HRV. **(A)** Mean HR of wolves and dogs when resting across conditions. **(B)** Mean HR of wolves and dogs when awake and inactive across conditions. **(C)** HRV of wolves and dogs when resting across conditions. **(D)** HRV of wolves and dogs when awake and inactive across conditions.

Overall, HR in wolves and dogs was lower when resting, as compared to being awake but inactive (Figures 1A,B). During rest, dogs in proximity of a social partner (human or conspecific) had lower HRs than when alone (Figure 1A and Table 3). In contrast, during inactive wakefulness, dogs’ HRs in proximity of a familiar human were higher than in the two other conditions (Figure 1B) and dogs’ HRs when alone or with conspecifics was similar. During rest, the HRs of wolves were lower in proximity to their pack members as compared to being close to a familiar human or alone (Figure 1A). In addition, wolf HRs seemed similar when resting alone or in proximity of a familiar human (Figure 1A and Table 3). When awake and inactive, wolf HRs near a pack member were lower as compared to being alone or close to a familiar human (Figure 1B). Furthermore, HRs of wolves and dogs were roughly similar when resting near their pack members whereas they differed in the two other conditions (Figure 1A): Dog HRs were lower than those of wolves when alone or close to a human partner (Figure 1A). During inactive wakefulness wolves had higher HRs than dogs when alone whereas in the social conditions the HRs of wolves and dogs were similar (Figure 1B). HRs also increased with age (estimate ± SE = 15.24 ± 2.40, χ^2^ = 14.65, *P* < 0.001) and body mass (estimate ± SE = 10.71 ± 3.59, χ^2^ = 5.19, *P* = 0.023), whereas sex and temperature had no significant effect (Table 3 and Supplementary Table 1).

**TABLE 3:** Descriptive statistics for the mean HR and RMSSD values.

			HR					RMSSD				
Species	Condition	Activity	Mean	SD	Min	Max	Median	Mean	SD	Min	Max	Median
Dog	Alone	Rest	66.60	9.81	55	81	63	357.00	180.94	59	530	415
Dog	Alone	Awake	76.43	12.80	64	117	75	254.28	152.96	31	453	315
Dog	Human	Rest	62.14	10.09	53	82	62	520.43	225.84	172	760	551
Dog	Human	Awake	80.67	10.33	63	95	81.5	210.50	128.53	55	416	188
Dog	Conspecific	Rest	57.71	8.03	47	70	59	594.57	113.53	408	746	615
Dog	Conspecific	Awake	76.57	14.73	61	102	73	339.71	222.21	103	627	230
Wolf	Alone	Rest	68.00	10.08	59	83	68	360.84	155.22	223	634	323
Wolf	Alone	Awake	87.67	23.15	61	94	84.5	298.00	189.20	30	504	308
Wolf	Human	Rest	72.00	8.12	63	85	70	241.60	126.68	60	359	251
Wolf	Human	Awake	79.40	6.23	69	85	81	132.00	98.94	43	267	85
Wolf	Conspecific	Rest	59.84	13.23	41	75	58.5	407.17	189.96	118	622	385
Wolf	Conspecific	Awake	76.50	18.81	60	105	67.5	262.17	185.56	16	530	236

*Descriptive statistics of dogs and wolves mean HR and RMSSD values grouped by conditions and activity.*

We found distinct inter-individual differences in HR in both dogs and wolves (see Supplementary Figure 1). In the dogs, two subjects seemed to drive the decrease in HR at rest, whereas two other animals had the highest HR during the pack condition. In the inactive but awake state, two individuals had the highest HRs in presence of a familiar human (Supplementary Figure 1A). Similarly, in the wolves, two animals displayed a higher HR when resting with a familiar human than when alone, whereas one female had a substantially lower HR in presence of a familiar human than when alone. Only one wolf had a higher HR when with her pack than when alone. During inactive wakefulness, two individuals had very high HRs when alone and for both of them the presence of a familiar human seemed to lead to a lower HR. Compared to the alone condition, all wolves displayed lower HRs in the presence of their pack mates; however, when compared to the human condition, two wolves had higher HRs and two lower HRs in the pack condition (Supplementary Figure 1B).

### Heart Rate Variability (RMSSD)

Overall, the full model was significant as compared to the null model (likelihood ratio test: χ^2^ = 40.15, *df* = 11, *P* < 0.001), i.e., species, activity, condition, or their interaction(s) affected the root mean square of successive differences (RMSSD, which expresses heart rate variability; HRV) in wolves and dogs. However, since the three-way interaction between species, activity, and condition was not significant (likelihood ratio test: χ^2^ = 3.47, *df* = 2, *P* = 0.18, Figures 1C,D and Table 3), we removed it from the model to explore the significance of the two-way interactions between our factors of interest: species, activity and condition. The two-way interaction between species and condition was significant (likelihood ration test: χ^2^ = 8.48, *df* = 2, *P* = 0.014); wolf and dog RMSSDs were similar in the alone condition, but in presence of a social partner dogs had higher HRVs than wolves (Supplementary Figure 2). Also, the two-way interaction between species and activity was significant (likelihood ration test: χ^2^ = 8.27, *df* = 1, *P* = 0.004), meaning that both wolves and dogs had similar RMSSDs when awake and inactive while at rest dogs had higher RMSSDs (Supplementary Figure 3). Finally, the two-way interaction between activity and condition was also significant (likelihood ratio test: χ^2^ = 7.99, *df* = 2, *P* = 0.018). When alone RMSSDs of wolves and dogs did not differ much between being awake or resting, whereas when with a social partner (human or conspecifics) RMSSDs where higher at rest than when awake (Figures 1C,D). RMSSDs decreased with age (estimate ± SE = −103.54 ± 41.19, χ^2^ = 5.07, *df* = 1, *P* = 0.024) whereas body mass, sex, and temperature had no significant effect on the RMSSD (Table 4 and Supplementary Table 2).

**TABLE 4:** Results for the HRV model.

	Estimate	SE	χ^2^	*df*	P^1^
Intercept	270.451	88.462			
Species (0: dog; 1: wolf)	80.431	133.413			
Human	–24.868	57.153			
Conspecifics	122.402	44.987			
Activity (0: awake; 1: rest)	149.889	43.636			
Body mass^2^	80.874	60.934	1.567	1	0.211
Temperature^2^	–5.988	14.578	0.162	1	0.688
Age^2^	–103.540	41.188	5.072	1	**0.024**
Sex (0: F; 1:M)	–68.764	69.425	0.973	1	0.324
Wolf:Human	–163.474	76.643	8.478	2	**0.014^3^**
Wolf:Conspecifics	–168.204	53.965			
Wolf:Rest	–130.650	42.885	8.266	1	**0.004**
Human:Rest	150.712	53.755	7.994	2	**0.018^4^**
Conspecifics:Rest	114.564	51.403			

*Significant p-value are in bold. ^1^Not indicated in the case where p-value had a limited interpretation. ^2^Predictors were z-transformed to a mean of zero and a standard deviation of one; original means (SD) were weight: 32.98 (9.49) kg, temperature: 22.41 (7.18)°C and age 2440.23 (801.93) days. ^3^Overall test of the two-way interaction between species and conditions. ^4^Overall test of the two-way interaction between activity and conditions.*

As the case with HR, we also found considerable individual differences in RMSSD. In two dogs it was overall lower than in the other dogs at rest. Also, individual dogs differed in their response to the presence of their pack; when resting two individuals had substantially lower RMSSDs close to their pack as compared to being alone, while during inactive wakefulness three individuals had higher RMSSDs with their pack as compared to the alone condition (Supplementary Figure 1C). At rest, two wolves, did not vary in their RMSSD, regardless of condition. During inactive wakefulness, the RMSSD of three wolves reached lowest values in the presence of the familiar human, whereas it peaked in two others in this condition (Supplementary Figure 1D).

## Discussion

Our results show than dogs and wolves’ cardiac output varies with degree of activity, social environment, and also quite substantially between individuals in the different contexts. Across all three conditions, both wolves and dogs were less aroused, showing lower heart rates (HR) and higher heart rate variation (HRV) when resting, as compared to inactive wakefulness. This aligns with previous results (Varga et al., 2018; Kortekaas and Kotrschal, 2019). However, in contrast to Kortekaas and Kotrschal (2019), dogs at rest were generally less aroused (i.e., had lower HR and higher HRV) than wolves but showed roughly the same cardiac parameters as wolves when awake and inactive. This discrepancy may be explained by the different social context in the two studies. Kortekaas and Kotrschal (2019) had an unfamiliar human filming all the experimental sessions, while in our study we controlled for familiarity by having either no human, a familiar human, or conspecifics nearby. The presence of an unfamiliar human could have been more arousing than soothing for the dogs than the presence of a familiar human. However, we also need to note that as we found a substantial individual variation in our data, the differences between the two studies might also be explained by small samples sizes, as discussed below.

Interestingly, we found that dogs at rest seemed to respond to the presence of a familiar human in a similar way as to the presence of their pack members (i.e., lower HR and higher HRV than when alone), whereas in our human-socialized wolves, pack members seemed to be more effective at being emotional social support than familiar humans. When dogs and wolves were awake, the differences between their cardiac parameters decreased, probably because both were more alert (increased HR and lower HRV) as compared to resting with eyes closed. More specifically, when awake and close to a familiar human, the HRs of our human-socialized wolves and dogs were similar, whereas the HRVs were still lower in wolves. Also, during inactive wakefulness, the dogs had distinctly higher HRs and lower HRVs in the presence of a familiar human than when alone or with pack members, whereas differences between conditions were less clear in the wolves. It seems that the presence of humans affected dogs differently depending on if the dogs were awake, where multiple other stimuli may influence them and reduce the effect of the human, or asleep when there were fewer external stimuli to distract the dogs. We suggest that when awake, the dogs anticipated interacting with the familiar human, which may have increased their arousal. Wolves showed similar arousal levels than dogs in the presence of familiar humans as indicated by similar HR but HRV in this condition was lower in wolves than in dogs. As HRV has been linked to cognitive processes (Maros et al., 2008; Luque-Casado et al., 2013), we speculate that the presence of familiar humans might have been cognitively more stimulating for the wolves than for the dogs. Alternatively, due to the presence of a close human partner, our socialized wolves could have also anticipated interesting events, such as a test situation, training session, or a social interaction, whereas the dogs may have responded with relatively unspecific excitement. In other words, our socialized wolves may attribute a different meaning or valence (HRV is frequently used to assess affective state; Kreibig, 2010) to the presence of a human than the dogs. Still, these wolves are similarly attentive to humans than the dogs (Range and Virányi, 2011), benefit from training interactions with them in a similar way (Vasconcellos et al., 2016), and interact socially with their hand-raiser (Ujfalussy et al., 2017). Our socialized wolves also differed in their HRV responses to the presence of a familiar person, which hints at the importance of the quality of social relationships and personality.

Rather than dogs being overall calmer than wolves due to domestication (Hare et al., 2012) or being “hypersocial” (vonHoldt et al., 2017), our results support the idea that pack members act as social support in wolves and that dogs use humans similarly as social support (Range and Virányi, 2015). This seemingly minor shift in the social significance of conspecific pack members versus socialized humans may have far-reaching implications. Support by a familiar human – in most cases the owner – can indeed help dogs to cope with a task or an unfamiliar situation (Topál et al., 1997; Gácsi et al., 2013; Horn et al., 2013). Comparable studies with human-socialized wolves are essentially lacking: Topál et al. (2005) found that, unlike 16 weeks old dogs, 16 weeks old hand-reared wolves did not show a preference for a human caretaker in an Ainsworth’s strange situation test. However, these wolves were not intensively in contact with their caretaker at the period the test was conducted (Virányi et al., 2008). Hence, as the wolves’ and dogs’ socialization substantially differed between research groups, results are hard to compare. Hall et al. (2015) found that socialized wolf puppies at 3, 5, and 7 weeks of age showed attachment behavior to a human caregiver. However, proper comparisons of wolves with dogs require similarly socialized and reared animals, as, for example, available at the WSC. A recent preprint, and hence not peer-reviewed study, comparing similarly reared wolves and dogs found that both species showed attachment toward their caretakers as adults (preprint; Wheat et al., 2020).

Our data also conforms to our daily experience with the WSC wolves and dogs; both show signs of attachment to familiar persons/their hand raisers and dogs tend to be generally more excited in the presence of such a person, while wolves behave in a relatively calm and focused way. Therefore, we suggest that the different ways dogs and wolves relate to humans as social partners also influences the way they cooperate with them but wolves and similarly raised dogs have probably more in common than they would differ in this respect. For example, in both wolves and dogs, attentiveness and willingness of the animal partners to cooperate seems to depend on relationship quality (Auer et al., 2011) and in both, successful cooperation generates a positive feedback on the social relationships between a human and a companion animal and reduces salivary cortisol (Vasconcellos et al., 2016). In fact, it has been demonstrated in a range of experiments that human-socialized wolves do cooperate with humans in a similar way than dogs (Range et al., 2019a, b) but subtle differences remain. For example, when given the choice, wolves tend to initiate and lead in such interspecies-cooperation, whereas dogs rather tend to follow the leading human and in general, the willingness of wolves to cooperate with humans seems to depend even more on relationship quality in wolves than in dogs (Range et al., 2019b).

Our data indicate strong potential effects of social relationships (with the human or the conspecifics present) as well as age, weight, and previous experience on the cardiac responses in the different contexts. This is not surprising, as individual bonds with both different humans and conspecifics differ in quality (Cimarelli et al., 2019). We suggest that this is an important underlying factor for much of the inter-individual variation found. In addition, personality is likely to be important. For example, during a safe haven test, reactive dogs (i.e., dogs prone to vocalizing when separated from their owner or growl and bark when approach by a threatening stranger) displayed HR and HRV changes during the test whereas the non-reactive dogs did not (Gácsi et al., 2013). Our moderate sample sizes in combination with relatively complex modeling did not allow us to include these potential causes of variability as factors but they should be kept in mind for future studies.

As age and weight may affect cardiac parameters, we will shortly discuss them here. In humans, HR generally increases in old age (Landowne et al., 1955; Umetani et al., 1998) but evidence for this in animals across their “normal” adult age range is rare, even more so in canids (Hezzell et al., 2013). As generally true for mammals, HR will decrease from puppyhood into adulthood in wolves and dogs, and may increase again in old age animals, mainly due to deteriorating health (Mosier, 1989; Strasser et al., 1997; Ferasin et al., 2010; Hezzell et al., 2013). Even less clear are the potential interactions between weight and cardiac responses in dogs. As HR in mammals is generally negatively correlated with body mass (Brody, 1945), this may also be true for dogs (Kirkwood, 1985; Sutter et al., 2007). However, most previous studies in dogs failed to demonstrate this (Ferasin et al., 2010; Lamb et al., 2010; Nganvongpanit et al., 2011; Rishniw et al., 2012). Hezzell et al. (2013) indeed found that HR scaled negatively with body mass, whereas Hamlin et al. (1967) reported that Great Danes HR frequencies exceeded that of miniature poodles. A recent study contributes to these contrasting results by reporting only a limited effect of body mass on HR (Cruz Aleixo et al., 2017). We presently controlled for body mass and age by adding them into the statistical model and found an influence of body mass on HR, while age affected both HR and HRV. Since wolves were heavier and older than the dogs in our study, the two variables could also have a confounding effect, e.g., if HR would increase with increasing age, this would also explain the body mass effect on HR. This is supported by Kortekaas and Kotrschal (2019), who also controlled for age and weight effects on cardiac output and found none. In their study, wolves were heavier but dogs and wolves were similar in age. Hence, in our study a confounding effect of age and weight is likely. We have no reason to assume a linear increase of HR with age over adulthood (Mosier, 1989; Strasser et al., 1997), the more as all our experimental animals were adults in good health, receiving regular veterinary care. Although we controlled for age and weight, we still found an effect of species, condition, and activity on HR and HRV. Hence, age and weight do not seem to explain much of the variability in our data. We therefore conclude that despite the differences in wolf and dog body mass and age, our comparisons of HR over different contexts are still valid. Such concerns do not affect HRV in a similar way as this parameter seems even more independent of body size or motor activity than HR (Cruz Aleixo et al., 2017).

We are aware that our relatively moderate sample sizes of six wolves and seven dogs, in combination with rather complex statistical models, do not allow us to draw final conclusion on the nature of wolves’ and dogs’ context-specific cardiac outputs. However, the cardiac parameters measured hint at a potential domestication-related difference in context-specific ANS modulation between wolves and dogs. Whether these results in our pack-kept dogs are representative also for pet dogs remains unclear but we suggest that the patterns we found are probably generic for human-socialized wolves and dogs and hence, would also be valid for pet dogs.

To conclude, wolves’ and dogs’ alertness and relaxation levels partially differed according to context. When resting, dogs more than wolves seemed to rely on human as social support, whereas when awake we measured similar cardiac responses to human proximity. This suggests that ANS modulation of dogs may be affected by domestication in a more complex way than suggested by simplistic interpretations of the selection-for-tameness hypothesis of domestication.

## Data Availability Statement

The raw data supporting the conclusions of this article will be made available by the authors, without undue reservation.

## Ethics Statement

The animal study was reviewed and approved by the institutional ethics committee at the University of Veterinary Medicine, Vienna, in accordance with GSP guidelines and national legislation (ETK-11/11/2018).

## Author Contributions

HJ-J, KKor, and KKot designed the experiments. HJ-J wrote the manuscript, collected the data, and analyzed them. KKor, FR, and KKot revised the manuscript. All authors have contributed to, seen, and approved the manuscript.

## Conflict of Interest

The authors declare that the research was conducted in the absence of any commercial or financial relationships that could be construed as a potential conflict of interest.
