# Supplementary material for: Context-Specific Arousal During Resting in Wolves and Dogs: Effects of Domestication?
Source: Front Psychol. 2020 Nov 24;11:568199. doi: 10.3389/fpsyg.2020.568199 (PMC7732590; doi:10.3389/fpsyg.2020.568199)
Supplement: Supplementary file 1 [file Data_Sheet_1.docx]

**
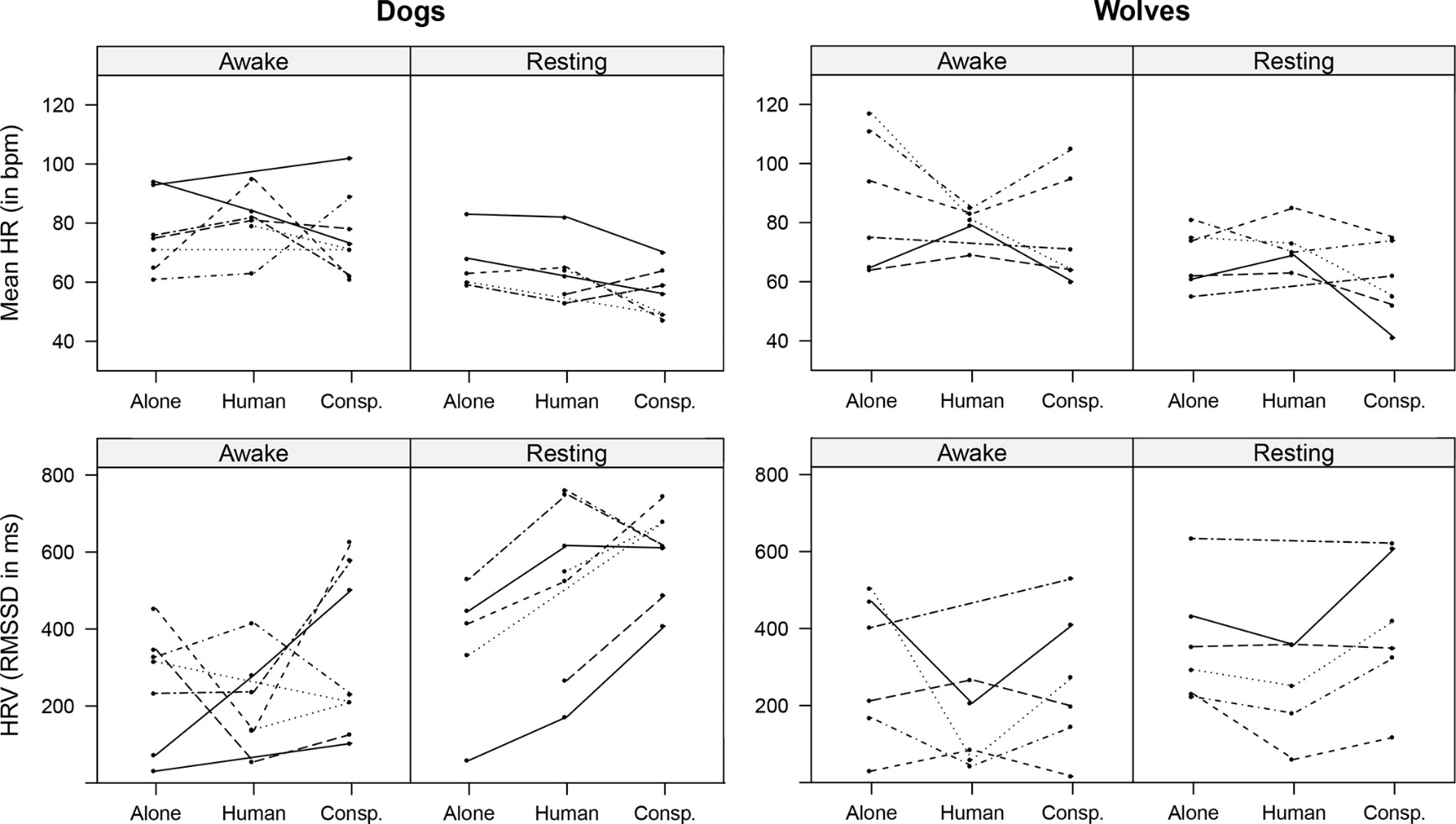
Supplementary Materials**

**Figure 1: Plot of the individual data**. A) Individual mean HR of dogs when resting or awake. B) Individual mean HR of wolves when resting or awake. C) Individual RMSSD of dogs when resting or awake. D) Individual RMSSD of wolves when resting or awake. Each line style represent a different individual.

**
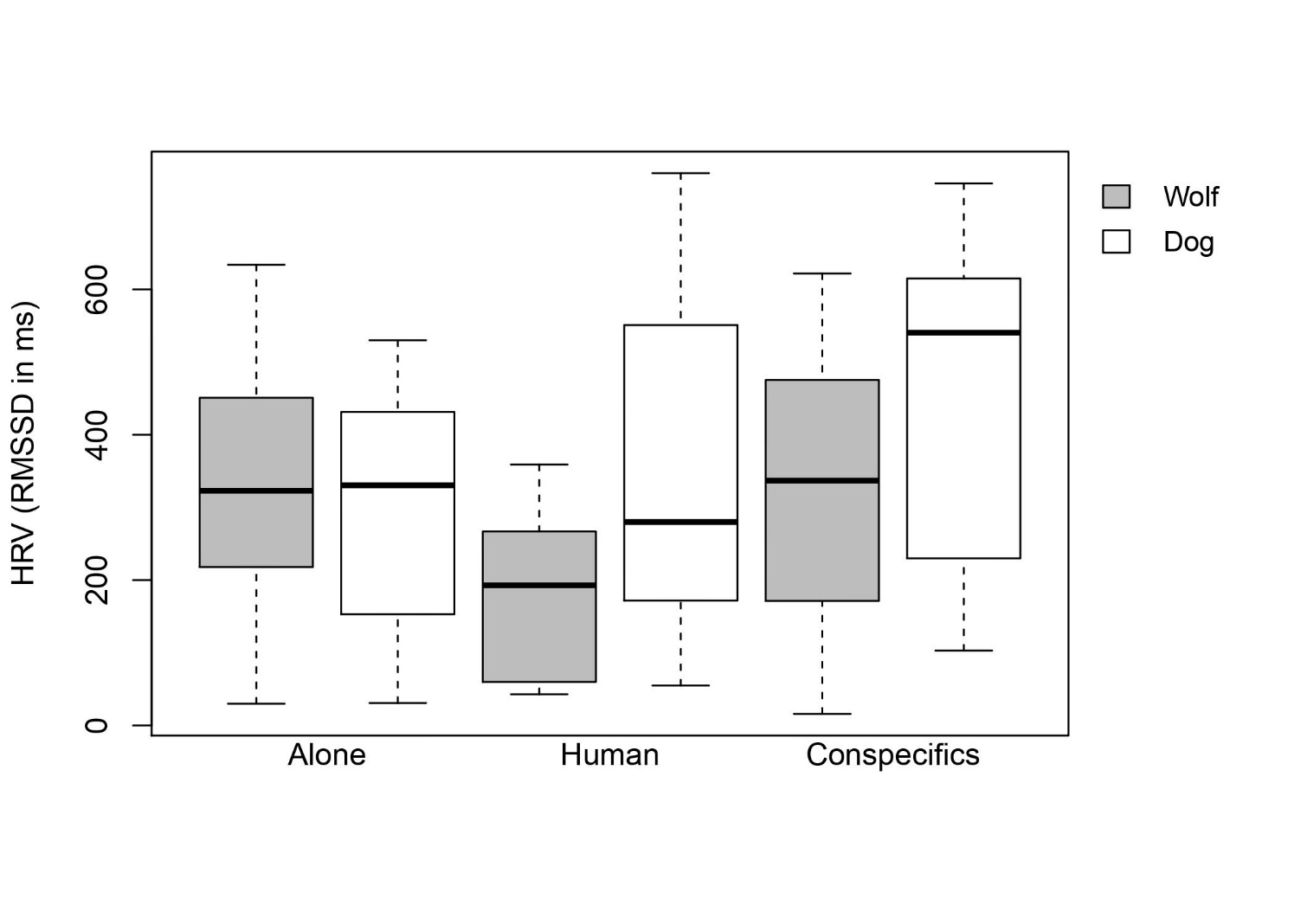
**

**Figure 2: Boxplot of HRV representing the interaction between species and condition.** The different level of the activity factor (resting or awake inactive) are both included in this boxplot.

**
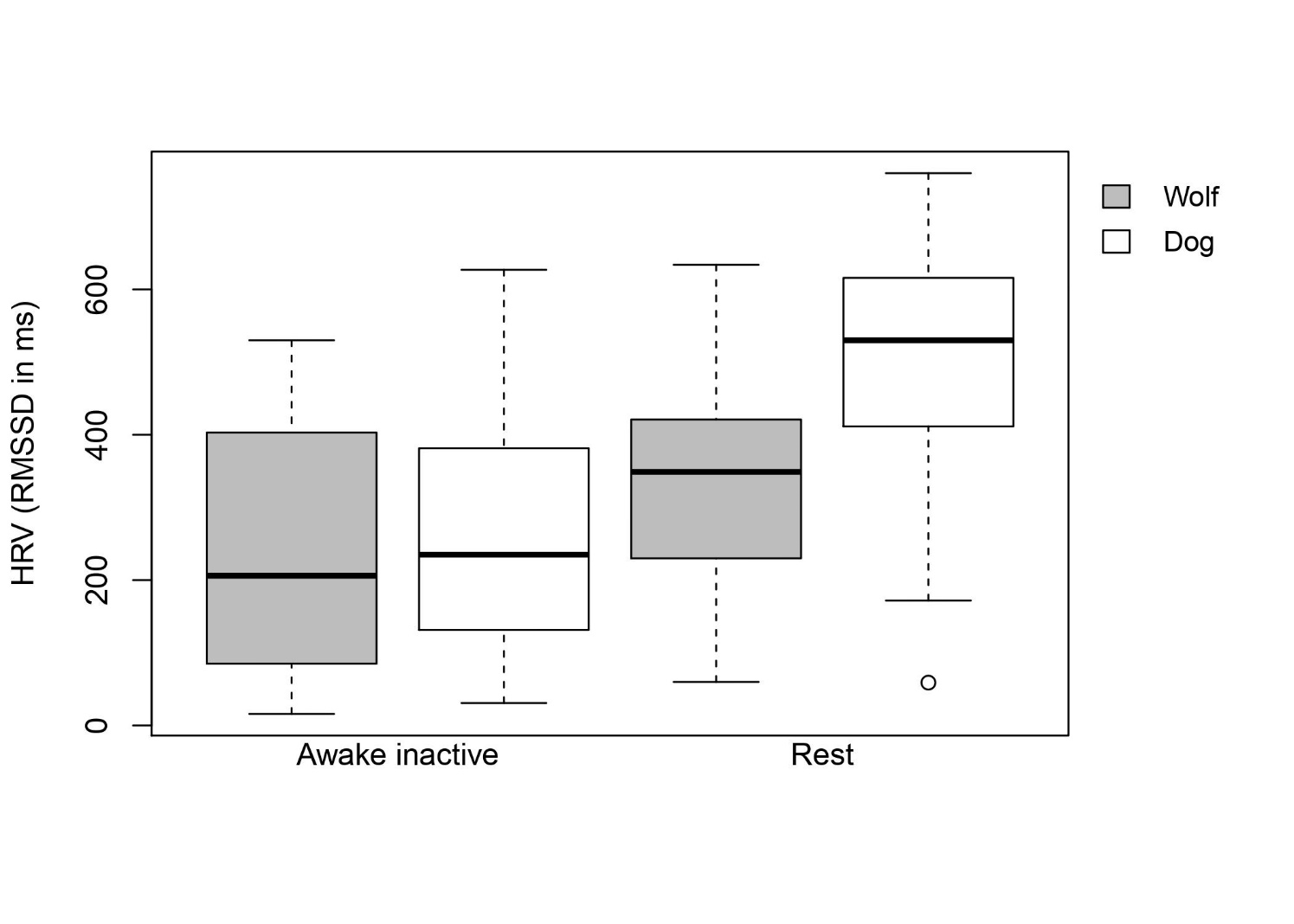
**

**Figure 3: Boxplot of HRV representing the interaction between species and activity**. The different level of the condition factor (alone, with a familiar human or with pack members) are all included in this boxplot.
