# Supplementary material for: Context-Specific Arousal During Resting in Wolves and Dogs: Effects of Domestication?
Source: Front Psychol. 2020 Nov 24;11:568199. doi: 10.3389/fpsyg.2020.568199 (PMC7732590; doi:10.3389/fpsyg.2020.568199)
Supplement: Supplementary file 2 [file Table_1.docx]

Supplementary Material

Table S1: Estimates, standard errors, lower an upper confidence interval and minimum and maximum values of the HR model

|  | Estimate | SE | Lower CI | Upper CI | min | max |
| --- | --- | --- | --- | --- | --- | --- |
| Intercept | 97.245 | 6.440 | 81.185 | 111.183 | 85.316 | 127.884 |
| Species (0: dog; 1: wolf) | -25.237 | 9.604 | -48.329 | 0.340 | -67.823 | -12.218 |
| Human | 9.908 | 4.123 | 2.100 | 19.327 | 6.189 | 13.864 |
| Conspecifics | 3.002 | 5.315 | -7.937 | 14.480 | -0.668 | 6.901 |
| Activity (0: awake; 1: rest) | -11.773 | 3.560 | -18.843 | -4.828 | -14.294 | -8.874 |
| Body mass^1^ | 10.706 | 3.585 | 1.190 | 19.384 | 7.685 | 29.470 |
| Temperature^1^ | -2.027 | 1.016 | -4.405 | 0.441 | -4.083 | -0.897 |
| Age^1^ | 15.248 | 2.402 | 9.001 | 20.828 | 6.113 | 23.909 |
| Sex (0: F; 1: M) | -5.251 | 2.470 | -11.713 | 1.534 | -11.107 | -0.461 |
| Wolf:Human | -21.823 | 6.053 | -34.864 | -9.783 | -26.241 | -16.480 |
| Wolf:Conspecifics | -16.729 | 7.465 | -32.717 | -1.932 | -20.688 | -7.496 |
| Wolf:Rest | -7.956 | 4.942 | -17.334 | 2.666 | -11.249 | -3.609 |
| C.human:Rest | -9.627 | 4.448 | -18.841 | -1.156 | -12.799 | -5.370 |
| Conspecifics:Rest | -7.030 | 4.361 | -15.656 | 2.223 | -10.718 | -4.177 |
| Wolf:Human:Rest | 22.889 | 6.490 | 9.816 | 35.788 | 18.818 | 26.666 |
| Wolf:Conspecifics:Rest | 10.030 | 6.185 | -2.276 | 21.923 | 4.199 | 13.718 |

^1^ predictors were z-transformed to a mean of zero and a standard deviation of one;

original means (sd) were weight: 32.98 (9.49) kg , temperature: 22.41 (7.18) °C and age 2440.23 (801.93) days.
