# Supplementary material for: Context-Specific Arousal During Resting in Wolves and Dogs: Effects of Domestication?
Source: Front Psychol. 2020 Nov 24;11:568199. doi: 10.3389/fpsyg.2020.568199 (PMC7732590; doi:10.3389/fpsyg.2020.568199)
Supplement: Supplementary file 3 [file Table_2.docx]

Supplementary Material

Table S2: Estimates, standard errors, lower and upper confidence intervals and minimum and maximum values of the HRV model

|  | Estimate | SE | Lower CI | Upper CI | min | max |
| --- | --- | --- | --- | --- | --- | --- |
| Intercept | 270.451 | 88.462 | 99.519 | 439.275 | -44.198 | 383.619 |
| Species (0: dog; 1: wolf) | 80.431 | 133.413 | -184.605 | 348.774 | -115.856 | 567.343 |
| Human | -24.868 | 57.153 | -148.773 | 88.136 | -56.304 | 19.503 |
| Conspecifics | 122.402 | 44.987 | 32.197 | 208.489 | 84.807 | 154.112 |
| Activity (0: awake; 1: rest) | 149.889 | 43.636 | 62.819 | 237.507 | 128.940 | 180.957 |
| Body mass^1^ | 80.874 | 60.934 | -39.359 | 207.593 | -153.117 | 173.482 |
| Temperature^1^ | -5.988 | 14.578 | -33.678 | 23.122 | -19.428 | 21.783 |
| Age^1^ | -103.540 | 41.188 | -187.868 | -21.468 | -137.475 | -73.401 |
| Sex (0: F; 1:M) | -68.764 | 69.425 | -197.447 | 62.918 | -151.351 | 51.167 |
| Wolf:Human | -163.474 | 76.643 | -319.109 | -16.261 | -193.285 | -128.575 |
| Wolf:Conspecifics | -168.204 | 53.965 | -280.68 | -62.354 | -219.126 | -143.187 |
| Wolf:Rest | -130.650 | 42.885 | -212.010 | -43.779 | -147.159 | -120.729 |
| Human:Rest | 150.712 | 53.755 | 45.197 | 259.557 | 117.884 | 184.879 |
| Conspecifics:Rest | 114.564 | 51.403 | 11.684 | 216.154 | 85.006 | 145.558 |

^1^ predictors were z-transformed to a mean of zero and a standard deviation of one;

original means (sd) were weight: 32.98 (9.49) kg , temperature: 22.41 (7.18) °C and age 2440.23 (801.93) days.
